# HOW DO FAMILY RELATIONSHIP PATTERNS IMPACT CAREGIVING BURDEN IN ADULT CHILD CAREGIVERS?

**DOI:** 10.1093/geroni/igae098.2415

**Published:** 2024-12-31

**Authors:** Jodi Heys, Jessica Strong

**Affiliations:** University of Prince Edward Island, Charlottetown, Prince Edward Island, Canada; University of Prince Edward Island, Charlottetown, Prince Edward Island, Canada

## Abstract

Living with dementia is not isolated to the person with the diagnosis. Family and friends play an integral role in providing support, advocacy, love, and informal care for a person with dementia. Spouses and adult child caregivers have a unique relationship with the person with dementia (PWD), and often report a different subjective experience of caregiving (Rigby, et al, 2019). Due to limited research on how family relationships pre-caregiving impact the caregiving experience, the goal of this study is to gain a better understanding of these complex dynamics, particularly for adult child caregivers. The current study looked at perceptions of cohesion, expressiveness and conflict in family of origin (Brief Family Relationship Scale), as well as anxiety (GAD-7), mood (PHQ-9), and caregiver experiences, including burden, overload, and competence, among others (Caregiver Reaction Scale) in a population of adults (28-68 years old, N=25). Preliminary analysis results indicate a relationship between family conflict pre-caregiving and caring overload (r =-0.49, p = 0.04), such that lower levels of conflict were related to lower levels of overload. Additionally, we found no significant correlations between earlier family patterns of cohesion or conflict with adult anxiety (Cohesion: r =0.15, p = 0.55; Conflict: r =-0.11, p = 0.65) or depression (Cohesion: r =0.05, p = 0.85; Conflict: r =-0.18, p = 0.50). We discuss the current findings in the context of family systems theory and child-parent relationships in adulthood.

